# Checklist and quality assurance tools for integration of two radiation oncology information systems (ROISs)

**DOI:** 10.1002/acm2.70435

**Published:** 2026-02-15

**Authors:** ByongYong Yi, Shafiq Ur Rahman, Shifeng Chen, Baoshe Zhang

**Affiliations:** ^1^ Department of Radiation Oncology University of Maryland School of Medicine Baltimore Maryland USA

**Keywords:** Integration, QA, radiation oncology information system (ROIS)

## Abstract

**Background:**

Merging two radiation oncology information systems (ROISs) is often necessary due to system changes or hospital integrations. ROIS integration is a high‐risk procedure, that requires clear procedural guidelines and comprehensive QA methods to ensure safe practice.

**Purpose:**

This paper presents checklists, procedures, and challenges associated with integrating a ROIS into a centralized system, providing procedural guidelines and QA methods. It also shares our experience of merging with one ROIS into another.

**Method:**

The integration process comprised five major components: machine information; under‐treatment patients’ information (treatment plans, history, images, and electronic medical records [EMR]); user‐generated workflows; ROIS user information; and beam‐related information, if any (e.g., beam calibration). The procedures were divided into three parts: site survey and preparation‐phase activities, QA during integration, and QA after integration. Software tools were developed to compare data before and after the merger. Integration of legacy data was not considered in this process.

**Results:**

We successfully integrated a standalone practice site into a main ROIS, which may serve multiple sites, over the course of a single weekend using the developed tools and checklists. By the following Monday, after 45‐person hours of integration work by therapists, dosimetrists and physicists, the newly integrated practice site was able to seamlessly use the centralized ROIS to continue radiation treatment for its patients already under care. The entire procedure was completed without any downtime at any site.

**Conclusion:**

We have developed and successfully tested a structured set of checklists, procedures, and tools for the seamless integration of a practice site into an existing ROIS. This approach provides the radiation oncology community with a framework for achieving safe and efficient practice integration.

## 1 INTRODUCTION

Radiation oncology information systems (ROISs) are often merged or migrated to other systems for various reasons: An institution may change its ROIS vendor, the ROISs of two hospitals under one management may merge, or a ROIS of a hospital may become part of a networked practice to maximize efficiency through cross‐coverage, resource management, patient safety, and standardized procedures. Site integration can also occur due to changes in institutional affiliation or business acquisitions by larger sites. In recent years, there has been a trend toward site integration and consolidation in the field of radiation oncology.^1^, ^2^, ^3^ This indicates that many cases of ROIS integrations may happen annually.

Integration of ROIS is a high‐risk process in radiation oncology practices. The scope of modern ROIS functions is extensive, ranging from treatment machine‐related databases to the control of treatment delivery, and from radiation oncology electronic medical records (EMR) to images and treatment history.^4^, ^5^ Errors during the migration process can lead to serious clinical consequences. Additionally, delays in integration procedures may impact scheduled treatments after migration. Therefore, the process must be carefully designed, seamlessly coordinated, and flawlessly implemented. Despite the importance and potential serious impact of the consequences, reports on the procedures or QA methods of ROIS integration have not been published to our knowledge.^6^

We will use the terms “main site” for the integrated environment and “community site” for the site merging into the integrated environment. To merge the community database (C‐DB) of the community site ROIS (C‐ROIS) with the centralized database (M‐DB) of the main site ROIS (M‐ROIS), integration checklists, procedures, and tools were developed to ensure smooth integration. This paper aims to share our experience on the technical aspects of ROIS integration. Items not related to patient treatment safety, such as billing, administrative, legal, and contractual details, are not discussed, as they are outside the scope of this report.

The dose calibration point, or the treatment machine coordinates of the community site may not be the same as those of the main site. For standardization, the main site should align these settings when merging ROIS, even though such adjustments are not inherently part of the ROIS integration process. In this report, these alignment efforts are also considered part of the merging process and are denoted as optional activities in the tables.

## 2 MATERIALS AND METHODS

### 2.1 Checklists for the site survey and the preparation phase

A site survey was conducted to collect site information and to assess any compatibility issues beforehand (Table 1). During the preparation phase, the machine models and clinical workflows of the newly merged practice were configured in the M‐ROIS. The above‐mentioned data were acquired during the consensus meetings described in Table 1, then the collected information was confirmed by another qualified medical physicist. Existing clinical processes, the necessity of modifying machine parameters, and the beam‐related information were reviewed to determine whether they should be maintained or changed according to departmental policy, particularly if differences existed between the community site and the main site.

**TABLE 1:** Check list for preparation phase.

Items	Tools	Timeline
**Site survey**: Collection of data of the institution of C‐ROIS for the preparation		
Vendors and versions of TPS, ROIS, HIS, EMR and treatment/simulation machines, and other related devices.	Manual	1 month before
Machine configurations such as definition of coordinates, calibration, modalities(photon/electron/proton), energies, etc.	Manual	1 month before
Communication and connectivity of all components	Manual	1 month before
In‐house/3rd party utilities	Manual	1 month before
Determine use of in‐house/3rd party utilities at M‐ROIS	Consensus meeting	1 month before
Treatment techniques or treatment programs (such as, VMAT/IMRT/3D, SRS, HDR, LDR, etc.)	Manual	1 month before
User accounts and their user rights	Manual	1 month before
Determine how the discrepancies of machine's configuration/definition and user right policies between two institutions will be processed.	Consensus meeting	1 month before
Determine which treatment techniques/programs existing in the (legacy) C‐ROIS should be commissioned in the (integrated) M‐ROIS.	Consensus meeting	1 month before
Estimate staff hours during migration by analyzing patient loads.	Consensus meeting	1 month before
Determine if a comprehensive set of machine commissioning is necessary for new TPS commissioning, if applicable	Consensus meeting	1 month before
Determine if a Patient ID system needs to be adopted for the C‐ROIS.	Consensus meeting	1 month before
**ROIS configuration**: Create a new institution to M‐ROIS		
Create a hospital entry for the community site.	ROIS apps	2 weeks before
Test the conversion of EMR document templates.	ROIS apps	2 weeks before
Test all the transferred clinical ROIS scripts.	ROIS apps	2 weeks before
Test transferred in‐house/3rd party utility tools.	ROIS apps	2 weeks before
Configure DICOM imports/exports for TPS, treatment/simulation machines and other related devices.	ROIS apps	2 weeks before
**Machines**: Create machine information to M‐ROIS and perform following		
Export treatment machine information from the delivery console of each machine and import it to the M‐ROIS. Check match between the exported/imported machine configurations	Treatment console and ROIS (if applicable) or manual	1 month before
If any of the configuration parameters (for example, definition of the coordinate system) need to be changed, special attention is required.	Manual	1 month before
Set up CT scanners or other imaging systems in the M‐ROIS system.	ROIS apps	1 month
Test and verify machine QA systems (including daily/monthly/annual) in the integrated system if applicable. For example, if the output factor is adjusted, re‐calibrate daily/monthly QA systems*.	QA systems	2 weeks before
**Connectivity and network configuration of the community site per new policy**		
Test interconnection including HL7 interface between HIS (hospital information system) of the community site and M‐ROIS if the HIS of the community site remains but not to be merged.	Manual	1 Month before
Configure network (including firewall and anti‐virus software) per the new institution policy.	Manual	1 Month before
**Patient Data Preparation**		
Check if patient ID needs to be changed per the requirements of the integrated ROIS system (M‐ROIS).	Manual	2 weeks before
Establish a plan to retrieve patient treatment data including CT/ RT‐Structure/ RT‐Plan /RT‐Dose /RT‐Treatment Record just after the C‐ROIS system is disabled for the migration.	Manual	2 weeks before
Establish a plan to retrieve EMR documents just after the C‐ROIS system site is disabled for the migration.	Manual	2 weeks before
(the output factor of new machine was adjusted) MU‐scale / re‐optimize all under‐treatment plans or re‐plan*. If needed, re‐do PSQA.	TPS or PSQA	1 week before
Establish / perform a dry‐run end‐to‐end plan for testing all the clinical processes during the migration (from simulation to final treatment delivery)	Manual	1 week before
**Staff**: To establish users to M‐ROIS per new policy		
Set up user rights for the clinical staff	ROIS apps	2 weeks before
Grant appropriate user rights for the clinical staff per the policy	ROIS apps	2 weeks before
Prepare / schedule / complete training of the clinical staff for the clinical staff from the community site.	Manual	2 weeks before
Implement the clinical procedures (including institutional practice guidelines) for the community site.	Manual	2 weeks before
Beam data and TPS: (Optional) If a TPS of M‐ROIS is to be used		
(Optional) Perform the commissioning of a newly configured machine at M‐ROIS. Depending on the situation, either comprehensive commissioning or a set of spot check may be sufficient. *	TPS	3 weeks before
(Optional) Check if the output factor of each linac needs to be adjusted per the policy or requirements of the M‐ROIS*, which differs to the C‐ROIS. *	Manual	1 month before
If the output factor of each linac needs to be adjusted, PSQAs of all cases are required after merging.	PSQA systems (either calculation‐based or measurement‐based)	Right after the output adjustment
(Optional) Import beam data into TPS and then commission each linac in TPS and verify dose calculation engines for each newly commissioned linac. *	TPS	2 weeks before

TPS: Treatment planning system, HIS: Hospital Information System, EMR: Electronic Medical Records, HL7: Health Level 7, ROIS: Radiation Oncology Information System, and PSQA: Patient Specific Quality Assurance, C‐ROIS: Community Site ROIS, M‐ROIS: Main site ROIS.

Machine names, treatment accessories, definitions of beam calibrations, machine coordinates, and other parameters were created in the M‐DB to follow departmental guidelines. Table 2 summarizes the checklist for the preparation phase.

**TABLE 2:** Summary of site survey.

	Community site	Main site	Notes
ROIS	Aria V15.6 (Varian, Palo Alto, CA)	Aria V15.6 (Varian, Palo Alto, CA)	C‐ROIS or the ROIS of the Community site stays as the legacy system
Machines	2 Linear accelerators, 1 HDR unit, 1 CT simulator, (Vender, machine model and other detailed information collected)		Information created at the centralized ROIS (M‐ROIS)
Hospital Information System	EPIC (Epic Systems, WI)	EPIC (EPIC Systems, WI)	The main site IT grants access to community site member.
Network	The community site IT helps the main site IT with network switch and firewall settings.	Both IT groups of the main hospital and the community site integrate the network of the community site into that of the main site.	
User accounts/rights	Microsoft active directory	Microsoft active directory	A new active directory account was created for each clinic staff of the community site.
3rd party utilities	None	None	
DICOM import/export	Export patient data by auto DICOM export in‐house tool; manual export through ROIS applications	Import patient data through ROIS applications	
Number of treatment patients of Community Site	30 per day		To determine how many hours for transferring, confirming and PSQAs.
Estimate staff hours during migration	Initially estimated 30‐physicist hours, 5‐dosimetrist hours, 7‐therapist hours, 12‐vendorhours and 12‐IT hours.		
Treatment Planning System *	Eclipse V15.6 (Varian, Palo Alto, CA)	Eclipse V15.6 (Varian, Palo Alto, CA)	Commissioning Data of Community Site moved to that of Downtown
Machine Coordinate**	Varian IEC Scale	Varian IEC Scale	No Need to change
Dose calibration Point***	Per TG‐51, SAD setup at 10 cm depth	Per TG‐51, SAD setup at dmax	Recalibration to SAD setup a dmax when merge

* Optional, NOT a necessary part of the ROIS merge, Commissioning data exported and reviewed,.

** Optional, NOT a necessary part of the ROIS merge, no need to change since using the same scale.

*** Optional, NOT a necessary part of the ROIS merge, Recalibration to match Downtown for safety and convenience.

During this phase, participants, specifying their scopes of work, verification procedures, and reporting methods. Each participant was required to review and understand their assigned tasks before the integration weekend to ensure that all migrations could be completed within the allotted time.

### 2.2 Checklist and procedures during integration

The integration process involved five major components: (1) machine reconfiguration, (2) transfer of information for patients under treatment, (3) integration of clinical processes and user scripts, if any, (4) user information, and optionally (5) dose calibration, beam calibration, and change of the machine coordinates. Appendices 1–5 further explain these five parts. EMR and DICOM data of the under‐treatment patients were exported to a shared network folder by in‐house automation tools and then were imported to M‐DB. It also was possible to export machine configurations, user scripts, beam data, and user information automatically since the migration was between two ROISs of the same brand. Comparisons between before and after each part of the integration were performed. Table 3 summarizes the procedure and the checklist for data integration.

**TABLE 3:** Procedure and its tools for data integration.

Items	Tools	Timeline
**Disconnection and Connection to ROIS**: Redirect the machine connection to M‐ROIS		
Confirm the completion of the last scheduled treatment by checking treatment history in the C‐ROIS.	ROIS apps or DB query	After the last treatment and before the migration
Extract a list of under‐treatment patients from the C‐ROIS.	ROIS apps or DB query	Same as above
Disconnect the C‐ROIS from the machines and users by the vendor engineers.	Vendor's apps	During migration
Verify DICOM Export/Import communication between the M‐ROIS and the machines.	ROIS's DICOM import/export apps or vendor's apps	During migration
Verify HL7 interfacing between HIS and the M‐ROIS.	Manual	During migration
**Patient Data Transfer**: Export/import under‐treatment patient data from C‐ROIS to M‐ROIS		
Export treatment information (CT, RT‐Plan, RT‐Structure, RT‐Dose, Treatment History) from the C‐ROIS.	AriaDicomRetriever^†^ or ROIS import/export apps	During migration
Export EMR documents from the C‐ROIS.	AriaDocRetriever^†^ or ROIS's EMR apps	During migration
Import the above treatment information into the M‐ROIS.	ROIS's DICOM import/export apps	During migration
Import the above EMR documents into the integrated EMR system	ROIS's EMR apps	During migration
**Confirmation of Patient Data Transfer**: Confirm the imported under‐treatment patient data by comparing the contents of C‐ROIS and M‐ROIS (Appendix 6)		
Retrieve patient demographic information and treatment scheduling from the C‐ROIS and the M‐ROIS. Then compare between these two ROISs’ contents.	PatientInfoTxParser^†^ or ROIS apps	During migration
Compare DICOM files (images, RT‐Plan, RT‐Structure, RT‐Dose, Treatment History) exported from the C‐ROIS and the M‐ROIS.	DicomComparator^†^ or ROIS apps	During migration
Compare EMR documents in Microsoft document format between the C‐ROIS system and the M‐ROIS system	WordParser^†^ or EMR apps	During migration
Compare EMR documents in PDF format between the C‐ROIS and the M‐ROIS.	PDFParser^†^ or EMR apps	During migration
Compare EMR documents and machine configurations in XML format between the C‐ROIS and the M‐ROIS.	XmlParser^†^ or EMR apps / ROIS apps	During migration
Compare blob^‡^ files (for example, such as CBCT projection files) between the C‐ROIS and the M‐ROIS.	HashComparator^†^ or ROIS apps	During migration
Confirmation of Machines: All machine information, configurations and Machine QA (Appendix 4)		
Machine information/configurations: such as Linac's series number, operating limits, treatment techniques, energies, add‐on accessory devices, coordinate system etc. Linac console allows this information to be exported into one or multiple xml files.	XmlParser or ROIS apps	During migration
(Optional) Match the machine configurations to M‐ROIS*	XmlPaser or ROIS apps	During migration
Output Verification and/or Recalibration (if needed)	Manual	During migration
Machine QA (Daily, Monthly, Selected Annual)	Manual	During migration
End‐to‐End Test: From CT scan to treatment delivery test		
CT scan of a phantom, planning, beam delivery and PSQA	Manual	During migration
(Optional) If TPS is newly commissioned, phantom PSQA with a spectrum of treatment techniques (VMAT, IMRT, 3D, Electron) is recommended. *	PSQA systems (either calculation‐based or measurement‐based)	During migration
PSQA: a group of representative under‐treatment patients with various treatment techniques and energies. If the machines are newly calibrated, or if TPS is newly commissioned, PSQA for all under‐treatment patients is desirable.	Same as above	During migration
Mode‐up tests for all the treatment plans of all under‐treatment patients in Linac's control console.	Machine's control console	During migration

† in‐house developed applications.

‡ blob: binary large object.

* Optional, NOT a necessary part of the ROIS merge, recommissioning, machine re‐configuration and/or recalibration to match Downtown for safety and convenience.

### 2.3 Post‐integration verifications and QA

Post‐integration verifications were performed immediately after the integration of the ROIS. These included an end‐to‐end test with a phantom patient, treatment mode‐up tests and patient‐specific QA (PSQA) for all newly calculated under‐treatment patients, since the outputs of the photon beams were changed. Regardless of changes to the definition of the output point, the PSQA remains an integral part of the end‐to‐end test. Unless the output changes, selected representative cases of PSQA were considered sufficient.

Exported DICOM files, machine data files and EMR files to the M‐DB were compared with the original files from the C‐DB using the methods described by Zhang et al.,^7^, ^8^ as summarized in Appendix 6.

To verify machine quality and establish a baseline, we performed weekly, monthly, and selected annual QA tests on the machines in accordance with institutional guidelines.

## 3 RESULTS

The integration process took about a month from the formation of the steering committee to its completion. The steering committee comprised of IT members, a physician, a nurse, a therapist, a dosimetrist, a vendor representative, and physicists.

Table 2 presents a portion of the site survey summary. Following the decision to transfer the commissioned beam data to the TPS of the M‐ROIS, a medical physicist devoted one week to thoroughly review all machine beam data, beam modeling, and machine configurations as part of the Preparation Phase. This process was akin to beam data commissioning for a new machine, excluding beam data measurements. The machines were newly registered in the M‐ROIS and verified by a qualified medical physicist.

Two weeks before the final integration, treatment plans for all under‐treatment patients were recalculated using the newly created beam model, since the definition of the dose calibration points, and machine parameters were made to match those of the main site. One week before the final integration, with vendor assistance, we performed end‐to‐end dry‐run tests (from simulation to planning to treatment delivery) to identify any potential glitches in the integration process. The vendor‐assisted dry‐run tests are described in Appendix 7. The IT group completed the network infrastructure, including both hardware and software, one week before the final integration.

Patient‐specific QAs for under‐treatment cases were redone after the TG‐51 output adjustment. These tasks were completed by two dosimetrists and five physicists during the integration weekend. If no calibration adjustment was required, one dosimetrist and one physicist would have been sufficient for a few selected cases of PSQA.

We initially estimated 35‐person hours of physicists and dosimetrists, and 7‐person hours of therapists for the migration weekend. However, it took 45‐person hours to complete all QA. Delays were mainly due to waiting for prerequisite tasks. PSQAs for all of the under‐treatment patients were performed to increase the confidence level after the new output adjustments followed by TG‐51. If the output adjustments were not required, it could be reduced to 30 person‐hours for physicists and dosimetrists. Vendor and IT staff spent 12‐person hours each, as estimated.

A vendor representative was part of the steering committee. Simultaneously, the vendor had its own workgroup responsible for vendor‐specific integration tasks, with a physicist representative and an IT representative of the radiation oncology department.

## 4 DISCUSSIONS AND CONCLUSIONS

It is essential to integrate all data carefully and to automate processes across all components whenever possible to reduce user‐entry errors. Vendors provide a certain level of integration services, but these are often limited. As shown in Table 3, we developed tools to export/import and confirm the fidelity of data transfer, such as AriaDicomRetriever, AriaDocRetriever, PatientInfoTxParser, DicomComparator, WordParser, PDFParser, XmlParser, and HashComparator. All these tools have been vigorously verified following the methods described by Moran et al.^10^ It is also important to consider a reasonable evaluation process as shown in Table 4. Toward the end of the 1^st^ day of the merger, the medical director and chief physicist may officially announce successful integration once all the evaluation criteria outlined in Tables 3 and 4 have been fulfilled.

**TABLE 4:** Procedure and its tools for 1st treatment day after data integration.

Items	Tools	Timeline
Fix any ROIS‐related issues by the onsite vendor's engineer(s)	ROIS apps	On the 1st treatment day
Fix any procedure‐related issues by clinical staff including physicists/dosimetrists/therapists	ROIS apps or manual	On the 1st treatment day
Declare success of integration by the medical director and chief physicist	Manual	At the end of the 1^st^ treatment day
Report of merge	Manual	When available

**FIGURE 1:**
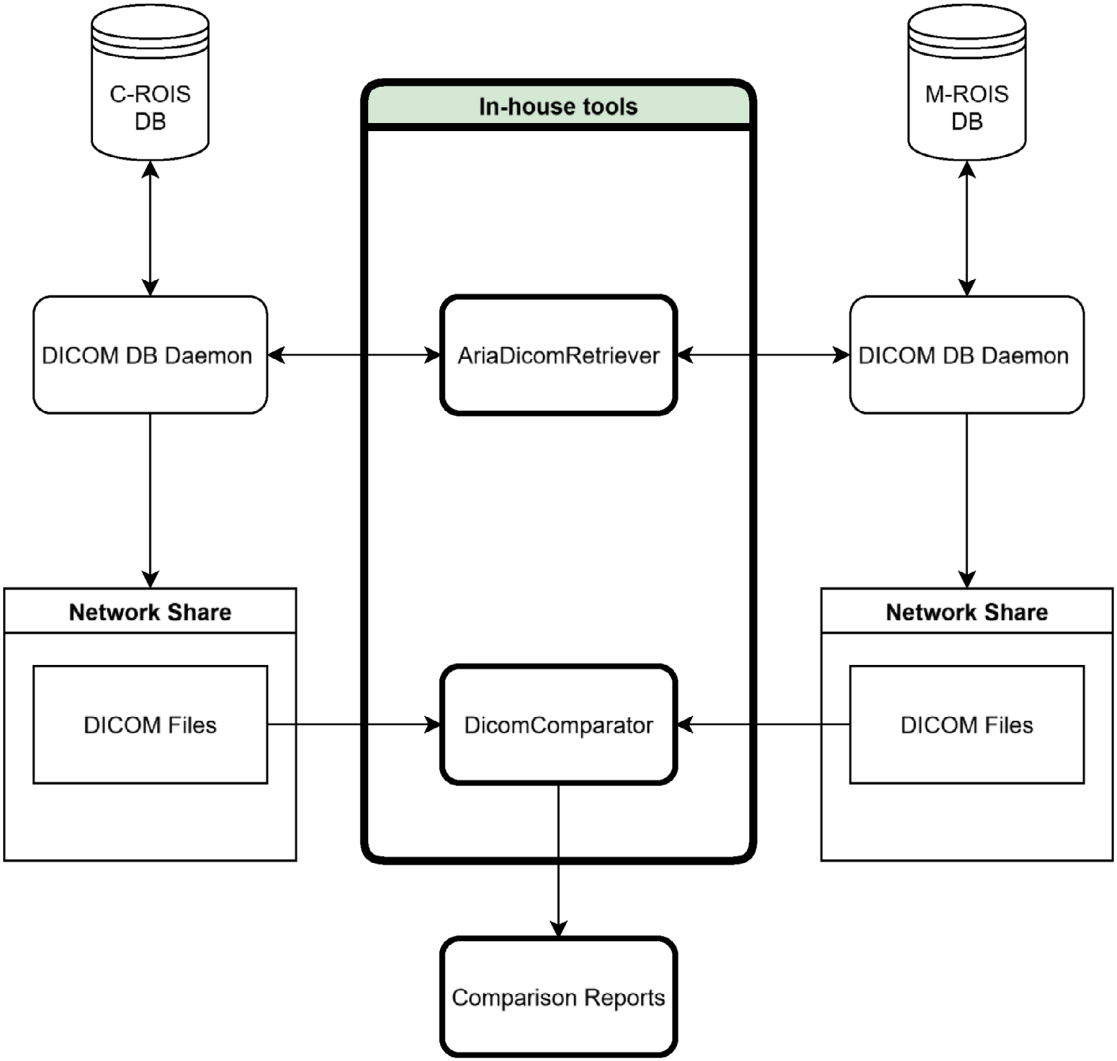
Schematic diagram of the export/import of DICOM files and their comparisons between the C‐ROIS (exported) and M‐ROIS (imported) database. The AriaDicomRetriever tool extracts CT images, RT‐Plan, RT‐Structure, RT‐Dose, and Treatment history files from both the databases. The DicomComparator performs content‐level comparisons of matched DICOM file pair across the two systems.

Figure 1 illustrates how the DICOM files are queried and exported/imported between two databases using one of the in‐house tools. The in‐house tool, AriaDicomRetriever, queries and imports CT, RT‐Plan, RT‐Structure, RT‐Dose, and treatment history data from both DICOM databases of the main and the community centers. Since the data in the M‐ROIS database is copied from the C‐ROIS database by the vendor and the hospital IT group, the contents are expected to be the same. Another in‐house tool, DicomComparator, verifies if the contents of both databases are the same. Other data such as medical documents, patient information, projection files of CBCT and machine information are retrieved and compared as the same patterns shown in Figure 1. The above information will be collected from both databases by PatientInfoTxParser and AriaDocRetriever then compared using PatientInfoTxParser, WordParser, PDFParser and XmlParser.

As described in Table 3 and Appendix 6, these tasks can be performed using the existing ROIS applications, albeit manually. The in‐house tools make the procedure faster and significantly less error prone. Table 3 lists the tools and their functions required for data integration.

It is crucial to verify all components, including machine energies, definitions of collimator, couch, gantry angles, beam calibration points, wedge factor points, and other dosimetric parameters, with the same rigor applied during commissioning.

The role of medical physicists in the integration task cannot be overstated. The physics group should lead the entire project from start to finish, from planning to final confirmation in the clinic. Their roles include leading technical integration and supervising other functional groups, such as vendors, dosimetrists, therapists, nurses, physicians, and IT staff. Comparisons of parameters and patient data before and after integration, along with dry runs and end‐to‐end tests immediately after the final integration, are pivotal to the success of safe integration.

Automation is known to reduce potential human errors^11^ and increase efficiency. For example, it took about an hour to export all data for 30 under‐treatment patients using in‐house automation scripts, compared to 15‐dosimetrist hours manually. It also took about an hour to verify all data, excluding time to write a merge report, using in‐house scripts, compared to an estimated 45‐physicist hours for 30 patients. This estimate does not include some parameter comparisons, such as MLC leaf positions of each control point of an intensity‐modulated radiation therapy plan, which we could compare using scripts.

It is essential to have a roll‐back plan in place in case the integration does not proceed as expected. For this reason, the C‐ROIS should be kept operational and unmodified until the integration is confirmed to be successful. If the integration does not proceed as planned, physicists, along with other clinical staff, should assess the situation and investigate the root causes. If the issues cannot be resolved within a predetermined time window, the team should initiate the roll‐back procedure to the C‐ROIS to ensure uninterrupted treatments. Any changes made to the C‐ROIS prior to and during the integration should be thoroughly documented to allow restoration to its original state if needed. Following a roll‐back, all patient data that had been imported into the M‐ROIS during the failed integration should be completely removed to maintain data integrity. With the roll‐back, since the C‐ROIS is expected to remain intact until the conclusion, no comprehensive QA is required. Only the end‐to‐end tests of testing cases and a selected monthly non‐radiation QA, such as the tests of the angles of gantry, collimator, and couch; the field sizes (X1, X2, Y1 and Y2), the couch coordinates and the MLC geometry will be sufficient. If machine calibration was performed, additional QAs, such as TG‐51 output measurements and PSQAs of selected cases, will be required.

The dose calibration point or the treatment machine coordinates at the community site may differ from those at the main site. While adjustments to align these settings are not required and are not formally part of ROIS integration, standardization is strongly recommended when merging two ROISs. In radiation oncology, standardization has been demonstrated to be the most effective means of improving patient safety and workflow efficiency, surpassing other approaches such as education, policies and procedures, and checklists.^12^ Accordingly, in this report, matching the coordinates and calibration point between institutions is regarded as part of the merging process.

The most challenging and important task is coordinating multidisciplinary teams within the limited integration timeframe, which we cannot overly emphasize.

We successfully connected a community site to the M‐ROIS without interruptions to clinical services or compromise of patient data using the checklist and QA tools developed in this study. The method suggested in this paper proved to be useful tools for ensuring a smooth transition from a C‐ROIS to an M‐ROIS system.

## AUTHOR CONTRIBUTIONS

**ByongYong Yi**: Conceptualization; data collection and curation; formal analysis; investigation, methodology; validation; visualization; writing—original draft; review and editing. **Shafig Ur Rahman**: data collection and curation; methodology; resources; validation; visualization; review and editing. **Shifeng Chen**: Methodology; resources; project administration; supervision; visualization; review and editing. **Baoshe Zhang**: Conceptualization; data collection and curation; formal analysis; investigation; methodology; project administration; supervision; resources; validation; visualization; review and editing.

## CONFLICT OF INTEREST STATEMENT

The authors declare no conflicts of interest

## Data Availability

DicomComparator is available at: https://github.com/zhangbs2000/DicomComparator

## DETAILS OF THE INTEGRATION PROCEDURES

### A.1 Machine re‐configuration

The treatment machines are usually situated behind a secure private network configured by the vendor. When the C‐DB is integrated with the M‐DB, the treatment machines and their firewalls must be reconfigured. These reconfigurations include setting up the private network/firewall and redirecting the machines to the M‐ROIS system. The vendor, with support from local IT infrastructure and network teams, should complete these reconfigurations. Additionally, there is a need to modify machine settings such as tolerance, technique, and treatment add‐ons. Qualified clinical physicists will verify these reconfigurations. We used our main site's model machine configurations to reconfigure the community site machines.

### A.2 Check list of under treatment patients’ data

During the integration phase, data for patients currently undergoing treatment were transferred to the M‐DB. These included treatment plans, images, structures, treatment doses, treatment history, patient demographics, and EMR documents. These treatment‐related information could be converted into DICOM files either manually or automatically. These DICOM files are subsequently exported to the M‐DB, again either manually or automatically. Similarly, EMR documents were exported to the main site. In‐house tools and scripts have been developed to facilitate the entire integration process. Data for patients who had already completed their treatments could be transferred at a later time.

### A.3 Clinical process integration

Clinical processes and user scripts identified for export during the Preparation Phase were exported according to their predefined priorities. During the Preparation Phase, we decided not to import any clinical processes or user scripts from the C‐ROIS. Instead, we adapted the existing clinical processes from the M‐ROIS. This includes, but is not limited to, all billing procedures and the hospital information system.

### A.4 Beam data integration

This is an optional task that applies only to either the transition of the planning system to that of the main site or the reconfiguration of the machine parameters such as the definitions of the machine coordinates and the beam calibration points. This task involves matching beam data among treatment machines, planning systems, and ROISs. The community site is equipped with two linear accelerators (Varian, Varian Medical Systems, CA), for which the M‐DB holds the beam data. Beam data were compared during the Preparation Phase. Due to differences in calibration points between the community site and the main site, calibrations based on AAPM Task Group‐51 were performed, followed by output adjustments for both machines.

### A.5 Migration of users

The community site was using the same type of ROIS as the main site. User access levels were modified before migration to align with those of the main site. Modifications in Active Directory, ROIS platform services, web services, and user profiles in ROIS were made to ensure that users could perform their day‐to‐day tasks smoothly. The list of users from the source database (C‐DB) was then exported to the central database (M‐DB).

### A.6 Data comparison

The DICOM comparison program confirmed the integration fidelity by performing brute‐force comparisons of all essential DICOM elements, ensuring the accuracy of patients’ images, structures, treatment plans, doses, and treatment delivery histories. For EMR documents, either a bit‐to‐bit comparison or a document parser was used to compare essential information. Our in‐house data comparison and parsing tools are built upon publicly available open‐source software packages, such as DicomPack (https://metacpan.org/dist/DicomPack) and XmlDiff (https://pypi.org/project/xmldiff/). We believe that each clinic can develop its own customized automation tools for seamless integration using these resources. PatientInfoTxParser uses the Microsoft Open Database Connectivity (ODBC) driver for SQL Server to query ROIS database and extract patient demographic information and treatment scheduling information. WordParser uses Apache POI (https://poi.apache.org/) to extract information from EMR documents in Microsoft Word. PDFParser uses pypdf (https://pypi.org/project/pypdf/) to extract information from EMR documents in PDF format. MD5 (Message Digest Algorithm 5) hash strings of files in proprietary formats were generated and the hashes before and after the migration were compared (HashComparator).

As a summary, retrieving DICOM and EMR documents can be retrieved either manually using the ROIS functions or automatically the tools described above. Hash code is used for files with proprietary formats, and the tool for the hash code generation is also described above. Comparisons of retrieved file are straightforward, except for DICOM file comparisons.

### A.7 Vendor‐assisted dry‐run tests

Prior to the dry‐run tests, the vendor assists in reconfiguring the Linac console to establish the network communication channel with the M‐ROIS. The reconfiguration process primarily involves the following steps: 1) modifying the firewall settings of the Linac's private network to permit outgoing connections to the M‐ROIS, 2) updating the ROIS destination to point to the M‐ROIS, and 3) reassigning the shared network drives between the M‐ROIS and the console computer. An existing test patient in the M‐ROIS is used to perform a series of checks, including plan mode‐up verification, beam delivery testing, treatment history verification, and IGRT image verification. Once all the planned checks are completed, the vendor must restore the original configuration in the console.
